# Systematic Review on the Metabolic Interest of Glucosinolates and Their Bioactive Derivatives for Human Health

**DOI:** 10.3390/nu15061424

**Published:** 2023-03-15

**Authors:** Antonio Costa-Pérez, Vanesa Núñez-Gómez, Nieves Baenas, Giuseppe Di Pede, Mariem Achour, Claudine Manach, Pedro Mena, Daniele Del Rio, Cristina García-Viguera, Diego A. Moreno, Raúl Domínguez-Perles

**Affiliations:** 1 Phytochemistry and Healthy Food Lab, Department of Food Science and Technology, CEBAS, CSIC, Campus Universitario de Espinardo-25, E-30100 Murcia, Spain; 2 Department of Food Technology, Food Science and Nutrition, Faculty of Veterinary Sciences, Regional Campus of International Excellence “Campus Mare-Nostrum”, Campus de Espinardo, University of Murcia, E-30100 Murcia, Spain; 3 Human Nutrition Unit, Department of Food and Drugs, University of Parma, 43125 Parma, Italy; 4 Human Nutrition Unit, Université Clermont Auvergne, INRAE, 63001 Clermont-Ferrand, France; 5 Microbiome Research Hub, University of Parma, 43124 Parma, Italy

**Keywords:** glucosinolates, breakdown products, metabolism, bioactivity, humans, clinical trials, epidemiological evidence

## Abstract

In the last decade, most of the evidence on the clinical benefits of including cruciferous foods in the diet has been focused on the content of glucosinolates (GSL) and their corresponding isothiocyanates (ITC), and mercapturic acid pathway metabolites, based on their capacity to modulate clinical, biochemical, and molecular parameters. The present systematic review summarizes findings of human studies regarding the metabolism and bioavailability of GSL and ITC, providing a comprehensive analysis that will help guide future research studies and facilitate the consultation of the latest advances in this booming and less profusely researched area of GSL for food and health. The literature search was carried out in Scopus, PubMed and the Web of Science, under the criteria of including publications centered on human subjects and the use of Brassicaceae foods in different formulations (including extracts, beverages, and tablets), as significant sources of bioactive compounds, in different types of subjects, and against certain diseases. Twenty-eight human intervention studies met inclusion criteria, which were classified into three groups depending on the dietary source. This review summarizes recent studies that provided interesting contributions, but also uncovered the many potential venues for future research on the benefits of consuming cruciferous foods in our health and well-being. The research will continue to support the inclusion of GSL-rich foods and products for multiple preventive and active programs in nutrition and well-being.

## 1. Introduction

To date, several studies have stressed the health-promoting benefits of cruciferous foods (Capparales Order, *Brassicaceae* fam. (Cruciferae)) [1] This family of vegetables comprises more than 3700 species, grouped into 338 genera, including model plants such as *Arabidopsis* [2]. Cruciferous plants are widely distributed around the world and constitute a family of vegetables, including species and varieties of socio-economic interest, such as *Brassica*, *Camelina*, *Crambe*, *Glaucocarpum*, *Raphanus*, *Sinapis*, and *Thlaspi* species [2]. Given this diversity, the wide variety of edible plant parts of cruciferous vegetables used depends on the species considered (e.g., broccoli or cauliflower inflorescences, cabbage leaves or Brussels sprouts, radish roots, mustard seeds, or oils obtained from canola, among others) [3]. Cruciferous foods are firmly rooted in Mediterranean culture and diet. The socioeconomic impact of these agri-food products is relevant in the international trade of highly-nutritional products [4]. Indeed, the health benefits obtained from the regular intake of cruciferous foods have been associated with their content of bioactive nutrients, as broadly reported elsewhere (micronutrients: amino acids, minerals and vitamins; macronutrients: proteins, dietary fiber, and carbohydrates), as well as phytochemical (non-nutrient) compounds (mainly represented by organosulfur compounds and (poly)phenols) [4].

With respect to the phytochemical composition of cruciferous foods, the epidemiological studies conducted so far have identified several glucosinolates (GSL) and their related hydrolysis products (isothiocyanates (ITC) and indoles) as their most characteristic bioactive derivatives responsible for their beneficial health effects. In this regard, to date, more than 130 GSL have been identified [5]. They are specifically distributed in the different members of the cruciferous family depending on several factors, namely species, cultivars, tissues, physiological stage, agro-environmental conditions during growth, and postharvest processing and storage practices [6]. Regarding the chemical features of GSL, they are water-soluble secondary metabolites responsible for several functions in higher plants (e.g., modulating plant pathogen and plant–insect interactions, among others) [5,7].

The biological properties of GSL are related to their particular chemical structure, which includes a common basic aglycone consisting of a β-D-thioglucose with a sulfonated oxime moiety and a variable side chain (R) derived from one of eight natural amino acids. Despite these common structural traits, only a few GSL have been evaluated for their bioavailability and metabolism within the frame of clinical trials and dietary interventions (Table 1). Based on the amino acid precursors, GSL are theoretically classified as aliphatic, mainly derived from methionine (Met), but also isoleucine (Ile), leucine (Leu) or valine (Val); indole GSL are derived from tryptophan (Trp), and aromatic GSL are derived from phenylalanine (Phe) or tyrosine (Tyr). Beyond this classification, Blazevic et al. (2020) presented a physiologically meaningful arrangement of GSL based on their type of degradation products: either stable or non-stable ITC or oxazolidine-2-thione. The first group was further subdivided into volatile/non-volatile ITC or hydrophobic/hydrophilic ITC, suggesting that this classification should reflect the biological problem analyzed [8].

**Figure 1:**
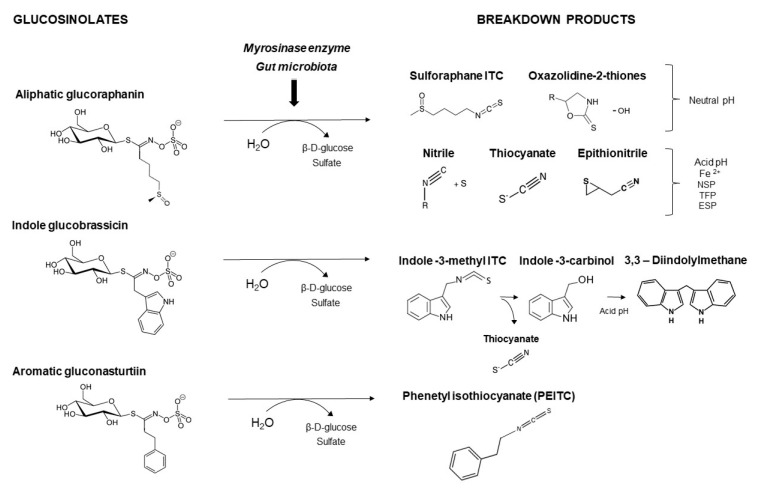
Scheme of the conversion of aliphatic, indolic, and aromatic glucosinolates to breakdown products by plant and intestinal myrosinase activity. ESP, epithiospecifier protein; ITC, isothiocyanate; NSP, nitrile specifier protein; TFP, thiocyanate-forming protein (Modified from Baenas and Wagner, 2019) [18].

GSL are stable compounds that need enzymatic reactions in order for them to be hydrolyzed and transformed into ITC and indoles [14]. The enzyme responsible for their hydrolysis is the so-called myrosinase (E.C3.2.1.147), a β–thioglucosidase located in specific vacuoles of plant cells named myrosin bodies. During plant processing or due to the transformation in the gastrointestinal system during digestion, the breakdown of plant tissues allows the release of GSL and myrosinase [9]. Then, the enzyme catalyzes the hydrolysis of thioglucoside, producing glucose and an unstable aglycone known as thiohydroxymate-*O*-sulfonate, which spontaneously derives into ITC, nitriles, thiocyanates, epithionitriles, and oxazolidine-2-thions, depending on the physicochemical conditions of the medium [14,15]. The formation of bioactive ITC is favored by a pH range between 6 and 7, while the hydrolysis of non-bioactive compounds takes place under acidic conditions and the presence of ferrous ions (Fe^2+^). Epithiospecifier proteins (ESP) and nitrile specifying proteins (NSP) favor the formation of nitriles and epithionitriles [16]. For GSL containing a β-hydroxylated side chain, the resulting β-hydroxy-ITC is unstable and spontaneously cyclize to form oxazolidine-2-thions, whereas the presence of an indole moiety drives the metabolization towards, for example, indole-3-carbinol (I3C) [10,17,18] (Figure 1).

Beyond the activity of the plant’s myrosinase, colonic microbiota can also hydrolyze GSL as a result of the activity of myrosinase-like thioglucosidases [19]. These transformation reactions define the actual concentration of the bioaccessible fraction of the ingested compounds in the intestinal lumen, which are prone to absorption in the small intestine and colon, thereby providing information on the intestinal absorption rate and the potential for biological activity [20].

From a physiological point of view, the bioavailability of bioactive compounds is defined as the fraction of a given compound that can be utilized by the body, depending on various processes, such as release from the food matrix, absorption, distribution, metabolism, and elimination [21]. The bioavailability of GSL and ITC depends on several factors, namely, the GSL content in plant tissues, the abundance and stability of myrosinase (the enzyme responsible for the hydrolysis of GSL towards the bioactive derivatives), the type of food (e.g., fresh vegetable), the type of processing (e.g., sanitation, blanching, cooking, etc.), and shelf-life conditions (e.g., frozen, room temperature, etc.) [22,23]. Other issues, such as the interactions of GSL with other food components and the reactivity of the formed ITC with other free amino groups and sulfhydryl side chains of proteins, may reduce their bioaccessibility [24], while dietary fiber can take part in the encapsulation of these compounds, thus delaying their intestinal absorption [25].

According to data reported in the literature on the bioavailability of organosulfur compounds, their intestinal absorption and systemic distribution are highly variable, ranging from 0.7% to 80.0% [26]. Regarding the digestion process of GSL and the hydrolyzed products, a small proportion is absorbed at the gastric level, while most of them reach the intestinal lumen. At this level, myrosinase could catalyze hydrolysis reactions. However, the currently available evidence indicates that, remarkably, most GSL reach the colon intact because myrosinase is usually inactivated during cooking processes or even denatured due to the pH of the gastric digestive juices [27]. In this regard, it should be noted that several microbial populations display myrosinase activity and can continue the hydrolytic reactions in the colon, thus helping to enhance GSL bioavailability and organic distribution, and consequently, biological potential [20].

The importance of reaching a high rate of bioavailability of GSL and their breakdown products is based on the biological functions attributed to these compounds. The biological functions referred to above have been attributed to their chemical diversity, being also highly dependent on the metabolic reactions that take place in the different cell types and tissues. In this regard, the formation of ITC and their subsequent conjugation with glutathione (GSH) is catalyzed by the enzyme glutathione *S*-transferase (GST), which allows the formation of a dithiocarbamate GSH-conjugate. Then, serial metabolic steps take place with the participation of the enzymes γ-glutamyltranspeptidase (GTP), ceramide glycanase (CGase), and acetyltransferase (AT) to finally form the mercapturic acid derivatives as the final products of the metabolic route, including ITC -cysteinyl glycine (-CYSGLY), -cysteine (-CYS), and -*N*-acetylcysteine (-NAC) conjugates. As an example, the ITC sulforaphane (SFN), derived from the aliphatic GSL glucoraphanin (GR), and its mercapturic acid-derived metabolites, have been widely used as biomarkers of broccoli consumption and in bioavailability studies. The SFN derivative SFN-NAC has been studied as the major metabolite found in urine, followed by SFN-CYS and SFN-GSH [28], while SFN-GSH and SFN-CYSGLY were the predominant compounds reported in plasma samples [29,30].

According to this diversity, obtaining updated knowledge on the circulating metabolites of GSL, ITC, and nitriles would allow the identification of the structure–function relationship for the array of biological activities referred to upon consumption of cruciferous foods. In this regard, although most reports have fixed the associated biological benefits to bioactive ITC and indoles, it cannot be ignored that some GSL and metabolic derivatives (e.g., progoitrin and thiocyanates from indole derivatives) could cause harmful effects when the dietary intake is excessive (e.g., >1 kg/day for several months). An excessive intake induces toxic and undesirable effects, such as goitrogenic processes [31]. Beyond this pathological issue, these vegetables have been blamed for thyroid hyperplasia, the reduced plasma level of the thyroid hormone, hepatic and kidney pathophysiology, or disrupted reproductive performance, among other deleterious effects [22]. However, these clinical entities have been associated with specific organosulfur compounds (e.g., thyroid enlargement with goitrin and derived ITC capable of blocking iodine uptake by thyroid cells that cause the inhibition of T4 hormone production [31]). Additionally, anemia symptoms have also been associated with compounds derived from *Brassicaceae*, as well as hepatic alterations, such as bile duct hyperplasia, megalocytosis, or hepatic necrosis and fibrosis, which have been related to nitriles formed as a result of GSL hydrolysis under specific pH conditions [32]. Thus, Brassicaceae-food-associated toxicity refers to an excessive daily intake of GSL that is not frequent in humans, although it is common in livestock, according to the specific composition of the diet. In this regard, it has to be mentioned that negative bioactivities due to the ingestion of Brassica foods within the frame of balanced diets, as demonstrated by the supply of sprout extracts in therapeutic quantities, have altered neither liver health indicators (transaminases) nor thyroid functions significantly or consistently [33]. Nonetheless, to discover the molecular origin of such undesired effects, the metabolomic analysis of the bioactive derivatives formed in humans needs to be further explored, as the correlation of the phytochemicals burden of these food matrices, with the actual positive effects on diverse pathophysiological conditions, has not been demonstrated yet [5]. This will also provide valuable information to define the molecular bases of the regulatory effects of GSL concerning metabolic diseases. Thus, these biological benefits have been analyzed recently, by resorting to 18 meta-analyses monitoring a population of >1.4 million participants. From this analysis, four major outcomes associated the dietary consumption of cruciferous vegetables with a positive evolution of diverse cancer types (such as gastrointestinal or pulmonary processes) [34,35,36,37]. However, the association with the clinical course and scope of additional pathologies, namely cardiovascular and coronary heart diseases, stroke, type 2 diabetes, or neurodegenerative and inflammatory processes, which has been reported as probable according to correlation studies in vivo [38,39,40,41,42,43], needs to be further studied for a more robust confirmation [44]. Despite this gap in knowledge, evidence has been reported on the capacity of Brassica foods to prevent type 2 diabetes (via the Nrf2 system represented by the range of antioxidant enzymes and the associated modulatory effect of the NF-κB inflammatory pathway), with respect to hyperglycemia and the level of pro-inflammatory molecules (e.g., TNF-α and IL-6), and the secondary damage induced by this pathology in a range of organs and bones [45,46]. These bioactivities are associated, for instance, with preventing risk factors for oxidative stress and neurodegenerative diseases via anti-amyloid and anti-secretase activity, although this activity has been mainly attributed to the content of (poly)phenols and flavonoids [47,48], which is not the focus of the present work.

In this scenario, the present article reviews the occurrence of GSL, ITC, and metabolic derivatives in humans after dietary ingestion of *Brassicaceae* foods, based on the evidence retrieved from nutritional and clinical research. The selection of data for the elaboration of this comprehensive review included different types of studies in healthy subjects, as well as in people presenting acute and chronic disorders, with increased attention given to the dietary sources of GSL and their relationship with bioavailability. Accordingly, the evidence summarized in the present review will allow future sound research on the health benefits of dietary sources of GSL and their respective bioactive derivatives, which will be responsible for describing the paradigmatic biological actions attributed to these foods, by also discussing the mechanisms of action already demonstrated.

## 2. Objectives

In the last decade, most of the evidence of the clinical benefits of including cruciferous foods in the diet, in different studies of pathophysiological problems, has been focused on the content of GSL in the source of compounds, and their corresponding ITC and mercapturic acid pathway metabolites, in addition to clinical, biochemical, and molecular parameters. To highlight the wide range of applications of these vegetables for improving consumer health and well-being, the present work aims at reviewing the relevant results of these leading works, which would contribute to guiding future research studies and facilitating the consultation of the latest advances in this booming and less profusely researched area of GSL for food and health.

## 3. Methodology

The selection of data was carried out under the criteria of including those publications centered on nutritional interventions with the use of Brassicaceae foods and different formulations, including extracts, beverages, and tablets, as significant sources of bioactive compounds, in different types of human subjects, and against certain diseases. The literature search was conducted in the Scopus^®^ (Elsevier, Amsterdam, Netherlands), Pubmed^®^ (National Library of Medicine, Bethesda, MD, USA), and Web of Science (Clarivate Analytics, London, UK) databases in May 2021 and updated in Jan 2022. The review protocol was executed following the recommendations of “Preferred Reporting Items for Systematic reviews and Meta-analysis (PRISMA)” [49]. The literature search was carried out by two authors and disagreements were solved by contrasting with a third author. The assessment of the risk of bias for each of the included studies was performed independently by the authors. The specific items revised were the study design (i.e., blinding, control and test groups), dietary source (i.e., food, extracts or derived formulas), and results of GSL derived metabolites by liquid chromatography (n = 3). Articles that did not meet these requirements were excluded of the present work. The syntax used for the search strategy was a combination of (“glucosinolate” OR “isothiocyanate” OR “indole”) AND (“bioavailability” OR “metabolism” OR “pharmacokinetics”), AND (human), AND (“urine” OR “plasma”). The period covered by the search was 2017–2021. The exclusion criteria included (i) studies that do not include information about metabolites from glucosinolates and isothiocyanates, and (ii) trial protocols. The final number of papers after refinement was 28.

The data compiled from clinical studies, including GSL and ITC analysis in biological samples, were organized in an standardized Excel file (from the FoodPhyt project, “A Healthy Diet for a Healthy Life”, JPI HDHL, 2019–02201), including several parameters for consultation: type of study, dietary source/food matrix, ingested dosages, description of the population (age, BMI, sex, ethnicity, and health status, either healthy volunteers or patients with any disease or physiological alteration), biofluid analyzed (plasma, urine, or ileal fluids), kinetic parameters (Tmax, Cmax, AUC, and time covered by AUC), type of compounds analyzed (family compounds, parental and derived metabolites, and their conjugates), the analytical technique used, and any reference or code of the study available in databases, and more specifically with a record in ClinicalTrials.gov Database (https://www.clinicaltrials.gov, accessed on 24 November 2021). The search results were incorporated into the PhytoHub Database (https://phytohub.eu, accessed on 24 November 2021) as a collaborative work under the framework of the JPI Foodphyt EU Project (2020 to 2024) (https://www6.inrae.fr/foodphyt/, accessed on 24 November 2021). The idea was to combine the data sets of health-related information and the studied bioactivity of these molecules at the clinical level, for directing further research on the human-centered health-promoting bioactivities of these compounds through the incorporation of cruciferous foods in dietary intervention and clinical research, to generate the necessary knowledge to demonstrate and support future allegations and recommendations for healthy living and well-being.

**Figure 2:**
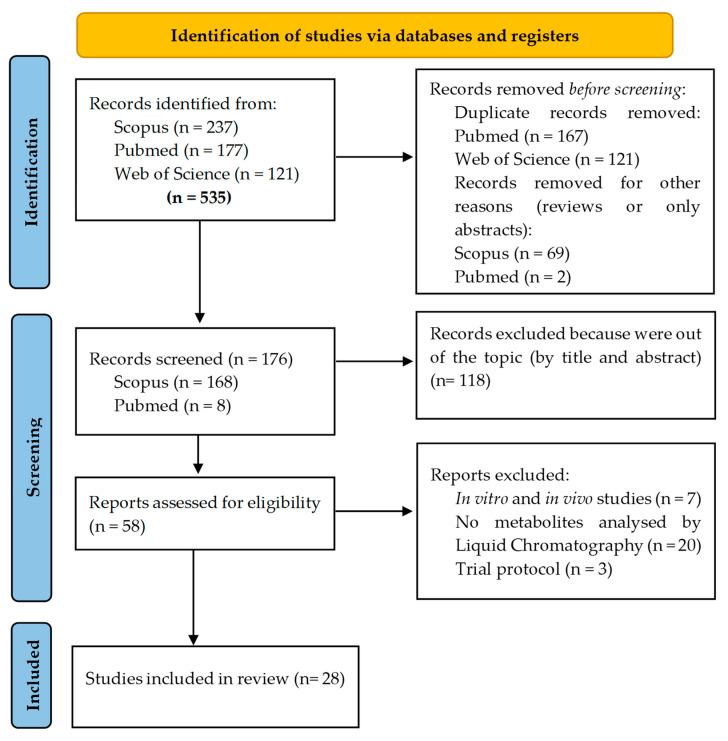
Flowchart of the study selection process.

The flow diagram for the study selection is shown in Figure 2. In the first identification phase, a total of 535 articles were obtained (237 from Scopus, 177 from Pubmed and 121 from Web of Science), of which 359 were removed (duplicates or other reasons). During the second screening phase, from the 176 papers identified as original, 118 were removed because they are out of the topic based on the title and abstract. So, a total of 58 articles were evaluated to determine their eligibility. In the last part of the screening, 30 articles were discarded in this phase because they are protocols, animal studies, in vitro cell culture studies and studies that do not include information about metabolites to ffinally render, the total of 28 papers included in the present review.

The revised studies were classified into three groups depending on the dietary source (i) Dietary interventions including the intake of cruciferous sprouts; (ii) Dietary interventions using mature cruciferous vegetables and derived products, and (iii) Seeds, extracts, or formulas enriched in glucosinolates. Tables 2–4 collect the specific information regarding the dietary source; dose of compounds consumed; type of biological sample; subjects characteristics; study record number in ClinicalTrials.gov Database (https://www.clinicaltrials.gov, accessed on 9 March 2023); concentration of metabolites excreted; analytical technique used; and reference identification number.

## 4. Dietary Interventions with Cruciferous Sprouts

This subsection includes the dietary interventions with cruciferous sprouts, as fresh germinating seeds and sprouts (a few days old) are naturally “functional” foods, rich sources of glucosinolates. Cruciferous sprouts with a high content of GSL, such as broccoli sprouts, are fresh foods that are becoming increasingly popular due to their contributions to the field of personalized nutrition and health. Dietitians and doctors widely recommend them because they are highly nutritious, low-fat, and low-calorie foods, with a great wealth of phytochemicals that promote healthy living. Likewise, the new consumption habits of current health-conscious consumers demand more foods with these characteristics to promote their well-being and physical condition [50]. To shed light on the actual bioactive compounds responsible for the beneficial effect attributed to these vegetables, when designing dietary interventions to study the bioavailability or functional traits attributable to individual compounds or the pool of bioactive phytochemicals present in a given food matrix, the first stage should be the comprehensive characterization of the edible material relative to the compounds of interest. Thus, when considering GSL and their breakdown products, cruciferous seeds are the main reservoir organ of the plant. Starting with the concentration reported at this stage, the content decreases during the emergence and development of the germinating seeds and sprouts, even though the content of GSL analyzed and reported in sprouts may reach values up to 10-fold higher levels than the content in mature inflorescences or aerial tissues [51]. Beyond the total content of GSL, when considering the different sub-classes, aliphatic GSL emerge as the group that contributes to the concentration recorded in seeds and sprouts to the highest extent, and their content is highly influenced by plant genetics. On the other hand, indole GSL are strongly modulated by environmental conditions but with some heritable variation [52,53].

In general, the evidence obtained on the bioavailability of GSL and derivatives after ingestion of cruciferous sprouts has boosted the recognition of the healthy characteristics of these plant-based foods [54]. This has encouraged the development of comprehensive studies of the actual value of these matrices as a source of bioavailable, bioactive compounds in vivo [55]. In this regard, 28 clinical trials were carried out between 2017 and 2021 to evaluate the bioavailability and metabolism of bioactive organosulfur after the consumption of cruciferous sprouts (mainly broccoli), also examining the capacity of these compounds and their metabolic derivatives to prevent specific markers of diverse pathological processes [56] (Table 2).

In these works, the analysis of the effect of a broccoli sprout extract (BSE) treated with myrosinase, on markers of diagnosis and prognosis of prostate cancer (PCa) in dietary interventions, led to the identification of various SFN and conjugated metabolites derived from the mercapturic acid pathway (SFN-GSH, SFN-CysGly, SFN-Cys, and SFN-NAC) in urinary and plasma fluids from 93 subjects aged between 50 and 78 years old. The treatment group received two BSE capsules daily (200 µmol SFN/day) over 4.4 weeks [57,58]. Only three individuals presented cumulative effects for SFN metabolites in the prostate tissues analyzed. This could be attributed to the short intervention period, the low doses administered, or concentrations lower than the limits of detection and/or quantification (LOD and LOQ, respectively) [59]. Indeed, the short duration or the low doses administered entail severe limitations for discerning the biological scope of the supplementation assayed [58]. According to the constraints identified in this study, longer interventions and possibly higher doses could be necessary to overcome these limitations [60]. In any case, based on the results retrieved, the authors concluded that there was a significant interaction between the treatment with BSE and the clinical course of PCa, as the prostate-specific antigen (PSA) decreased by 50% in most patients, despite the variability in the gene expression patterns associated with the treatment. Thus, SFN was identified as a candidate chemical inhibitor for controlling the expression of genes involved in PCa development, which are currently treated with immunotherapy. Moreover, the assessment of the molecular mechanisms responsible for these biological benefits, specifically at the transcriptomic level, indicated a 4.3-fold lower level of long non-coding RNA1, which is regulated by the androgen receptor (*ARLNC1*) in patients with PCa when treated with BSE, as well as a 7-fold decrease in alpha-methyl acyl-CoA racemase (*AMACR*) mRNA levels.

Interestingly, these findings were not associated with a significant decrease in histone deacetylase (HDAC) activity or associated biomarkers in the prostate tissue. However, as mentioned before, the biopsies from only three participants showed detectable levels of SFN, with no patient demographics or urine/plasma metabolite levels found to correlate with the concentration of SFN. Based on this premise, it can be suggested that overcoming the constraints of the experimental design to achieve higher and more consistent concentrations of the bioactive compounds (SFN) would allow covering the gap in the effect of these compounds on the biological changes observed. Despite this limitation, high levels of SFN metabolites were found in plasma and urine, with a transient decrease in HDAC activity at 3 h after consumption, and an increase at 12 h. This observation is corroborated by the description of the capacity of SFN to significantly reduce *AMACR* expression in Caco-2 cells in vitro, which is involved in colon cancer, suggesting that dietary SFN may effectively contribute to reducing the expression of some genes critically related to cancer [58]. This completes the previous information provided by Castro et al., who described the capacity of SFN to decrease the expression of specific oncogenes, such as *CR1* and *CRIPTO-3/TDGF1P3* in breast cancer [61]. Apart from this, it has recently been described that the modulation of gene expression by SFN includes both upregulation and downregulation of the expression of a range of oncogenes (the expression of 11 oncogenes was modified significantly). Thus, colon cancer patients had an increased expression of *TIMP1*, *CCL20*, *SPP1*, *AURKA*, *CEP55*, *NEK2*, *SOX9*, and *CDK1*, or a downregulation of *CRYAB*, *PLCE1*, *MMP28*, *BMP2* and *PLAC8*. According to these results, it was observed that SFN chemoprevention should be considered carefully, depending on the cancer phenotype and the genes involved in each specific type of cancer [62].

**Table 2:** Dietary interventions including the intake of cruciferous sprouts.

Dietary Source–Chronic or Acute Intake	Sulfur-Nitrogen-Based Compounds-Dose		Biological Samples	Subjects Characteristics	Study Record Identifier (NCT Number)	Metabolites and Conjugates–Concentration Range		Analytical Technique	Reference
Myrosinase-treated broccoli sprout extract 8 weeks	GR 200 µmol sulforaphane/day		Plasma and urine	Men prostate cancer risk: aged 65.7 ± 5.4 years who were scheduled for prostate biopsy	NCT01265953	SFN: 0.0001/0.66 µM (plasma/urine)		LC-MS/MS	[58]
						SFN-GSH: 0.03/0.0002 µM			
						SFN-CysGly: 0.04/0.005 µM			
						SFN-Cys: 0.02/1.23 µM			
						SFN-NAC: 0.03/2.9 µM			
Beverages of Broccoli sprout powders (High, medium, low dosage) Nightly, 10 days	GR and SFN 100 mL of beverage/day 120–600 µmol GR 8–40 µmol SFN		Urine	Healthy adults (24–65 years old)	NCT02656420	SFN (Mercapturic acid benzene deriv.)	0.67–1.09 nM	LC-ESI-MS/MS-selected reaction monitoring (SRM)	[63]
						SFN-Cys			
						SFN mercapturic			
Broccoli sprout powder 1 day	GR 1 g of broccoli sprout powder		Urine	5 healthy adult participants (4 males and 1 female, between the ages of 40 and 50 years)	N.A.	ITC metabolites: 6.6 (powder), 8.2 (gel-cap), and 4.7 (enteric-cap) µM		HPLC-UV-VIS/DAD	[64]
Broccoli sprouts 10 weeks	GR 30 g broccoli sprouts/day		Urine	Healthy obese adults (35–55 years old)	NCT03390855	SFN: 0.543 µM (day 70)		UHPLC-QqQ-MS/MS	[65]
						SFN-Cys: 0.8 µM			
						SFN-NAC: 2.301 µM			
						3,3′-DIM: 0.707 µM			
Broccoli sprouts 5 weeks	GR 30 g broccoli sprouts/ day		Urine	Healthy obese adults (men, non-menopausal women, and post-menopausal women; 40–60 years old)	NCT03390855	SFN: men 0.4604; pre-menopausal 0.4989; post-menopausal 0.8937 nmol/mg creatinine (day 35)		UHPLC-QqQ-MS/MS	[66]
						SFN-Cys: men 0.466; pre-menopausal 0.3727; post-menopausal 1.8191 nmol/mg creatinine			
						SFN-NAC: men 1.5031; pre-menopausal 1.8079; post-menopausal 4.0647 nmol/mg creatinine			
						3,3′-DIM: men 0.7186; pre-menopausal 0.7467; post-menopausal 0.5544 nmol/mg creatinine			
*Brassica carinata* sprouts (AVRDC) 1 day	Epithionitrile 15.2 g *B. carinata* sprouts		Urine	Healthy adults	N.A.	*N*-acetyl-S-(3-cyano-2-(methylsulfonyl)propyl-cysteine 3 h: 37 µM		UHPLC-ESI-(Q)ToF-MS	[67]
Broccoli sprouts powder drink 24 days	GR or SFN (800 and 150 µmol, respectively)		Urine	Healthy volunteers	NCT01008826	Day 6, TTA: 3.4/1.68 µmol/24 h (from GR/SFN matrix)		UHPLC-QqQ-MS/MS	[68]
						Day 6, GR: 3.71/ND µmol/24h			
						Day 6, SFN: 19.22/103.22 µmol/24 h			
Broccoli sprouts powder 1 day	GR	0.64 g broccoli sprouts powder	Urine	Five participants (two males, and three females, aged 22–52)	N.A.	SFN: 2.06 µM		LC-MS/MS	[69]
	Glucoiberin					Iberin: 0.23 µM			
Broccoli sprouts powder with inactive myrosinase 1 day	GR	0.64 g broccoli sprouts powder	Urine	Five participants (two males, and three females, aged 22–52)	N.A.	SFN: 0.66 µM		LC-MS/MS	[69]
	Glucoiberin					Iberin: 0.12 µM			
Gel capsule: oral formulation extracted from broccoli sprouts 28 days	GR 50, 100 or 200 µmol SFN		Plasma	17 patients (Caucasian descent), 12 female and 5 males, 22–66 years old (mean 47), with skin melanoma	N.A.	SFN: 120 ng/mL (range 1–208 for the 50 μmol group), 206 (range 89–420 for the 100 μmol group), 656 ng/mL (range 396–1305 for the 200 μmol group)		LC-MS/MS	[70]
Gel capsule: oral formulation extracted from broccoli sprouts 28 days	GR 50, 100, or 200 µmol SFN		Skin	17 patients (Caucasian descent), 12 female and 5 males, 22–66 years old (mean 47), with skin melanoma	N.A.	SFN: 0 ng/g (range 0–21.8 for the 50 μmol group); 0–18.9 ng/g (range:0–18.9 for 100 μmol group); 34.1 ng/g (range for the 200 μmol group)		LC-MS/MS	[70]

Abbreviations: 3,3′DIM: diindolylmethane; GR: glucoraphanin; HPLC-UV-Vis/DAD, high-performance liquid chromatography coupled to ultraviolet-visible and diode array detectors; ITC: isothiocyanates; LC-MS/MS, liquid chromatography coupled to mass spectrometry; MRM, multiple reaction monitoring; N.A.: not available; SFN: sulforaphane; SFN-Cys: sulforaphane cysteine; SFN-CysGly: sulforaphane cysteinyl glycine; SFN-GSH: sulforaphane glutathione; SFN-NAC: sulforaphane *N*-acetylcysteine; TTA: 2-thiothiazolidine-4-carboxylic acid; UHPLC-ESI-(Q)ToF-MS, ultra-high performance liquid chromatography coupled to electrospray ionization and mass spectrometry with the quantitative time of fly; UHPLC-QqQ-MS/MS, ultra-high performance liquid chromatography coupled to mass spectrometry with triple quadrupole technology. NCT Number or record study number assigned in ClinicalTrials.gov.

In order to determine the effective reference dose of a broccoli sprouts beverage for detoxifying carcinogenic air pollutants (benzene), Chen et al. administrated a drink enriched with glucoraphanin (GR) and SFN from 3-day-old broccoli sprouts to healthy adults. The researchers focused on the excretion of the metabolites SFN-NAC, SFN-CYS, and non-esterified SFN, which represent 80–81%, 12–14%, and 5–7% of the total SFN forms, respectively. This excretion percentage did not change during the intervention, indicating that the bioavailability remained constant. The enhanced excretion of the urinary biomarker of benzene detoxification *S*-phenylmercapturic acid (SPMA) was measured in the urine collected every 12 h during the 10-day intervention. Out of the 132 samples analyzed, >95% had detectable concentrations of SPMA, being significantly increased after consumption of the high dose of beverage (600 and 40 μmol GR and SFN, correspondingly), suggesting that consumption of >10 μmol SFN per 24 h may represent the lowest effective dose of the BSE affecting this biomarker. In this sense, the authors estimated the equivalency of the consumption of a broccoli sprouts beverage as compared to a portion of market-stage broccoli florets (60 g, ~1 cup) in ~25 μmol of SFN metabolites excreted in 24 h, considering 2 μmol/g of GR in broccoli at the market stage, and a theoretical conversion/excretion yield of 20% [63].

Another interesting study on the bioavailability of organosulfur compounds compared the absorption of SFN from BSE (1 g) administered in water or formulations with coated and uncoated gelatin capsules. The amount of total SFN metabolites excreted was higher for broccoli sprouts powder and uncoated capsules (18–24 µmol) as compared to coated capsules (8.5 µmol), suggesting a slow formation of ITC by myrosinase. This was due to an incomplete dissolution of the enteric-coated capsules in the intestine. In addition, the maximum peak of SFN excretion appeared at approximately 3.7–3.9 h after ingestion of broccoli sprouts powder and uncoated gelatin capsules, with almost all ITC excreted in urine after 12 h, while the enteric-coated capsules showed a delay in the urinary excretion of SFN (mean time of 15 h). Such constraints may be overwhelmed by using the multi-unit granule system (MUPS), since the small size of the granules would increase the interaction with the surface of the intestinal tract for drug absorption, thus allowing for a faster dissolution. Finally, the authors showed an upregulation of the transcription factor nuclear factor erythroid 2-related factor 2 (Nrf2) in lymphocytes cultivated in vitro, but not in lymphocytes isolated 3 h after broccoli sprouts intake by the volunteers, which may be explained by the low concentration of ITC found in plasma (1 µM) [64].

The anti-inflammatory capacity derived from GSL intake focusing on overweight individuals (related to a chronic inflammatory state) was evaluated by an intervention study where volunteers consumed an average of 51 mg GR and 20 mg of neoglucobrassicin (NeoGB) daily for 10 weeks, both of them from broccoli sprouts [65] (Table 2). The relevant effect obtained was a slight decrease in body fat mass after the intervention study. Moreover, the plasma concentrations of the pro-inflammatory mediator of the immune response, interleukin-6 (IL-6), decreased significantly (by 38%) after 70 days of daily broccoli consumption, remaining in low values for the next 20 days (until day 90). Nonetheless, after the end of the dietary intervention, the levels of IL-6 in peripheral blood plasma partially returned to their basal concentration. This suggests that the dietary intervention should continue, in order to maintain the modulatory effect on pro-inflammatory interleukins. A decreasing effect was also observed on the C-reactive protein produced in the liver in response to inflammatory processes. The metabolite 3,3′-diindolylmethane (3,3′-DIM), derived from indole GSL intake, was detected in all urine samples. In turn, SFN metabolites were formed via mercapturic acid; the one found in greater quantity was SFN-NAC, while the unesterified form of SFN exhibited the lowest excretion. The percentage of individuals in whom significant increases of urine SFN-NAC were detected during the intervention study relative to the basal concentrations was 45%. A similar trend was observed for the metabolites SFN-CYS and unesterified SFN, which increased by 67.5% and 82.5%, respectively, as a result of the intervention. This work showed that broccoli sprouts, which present a GSL concentration 5 or 10-fold higher than the mature broccoli heads [71], can modulate IL-6 and C-reactive protein levels, attenuating, therefore, chronic inflammation [65].

In another study, the highest levels of 3,3′-DIM in urine were detected on day 35 in postmenopausal women. This study was carried out with 69 healthy overweight subjects allocated into three groups (men, non-menopausal women, and post-menopausal women), who consumed broccoli sprouts (30 g/day, mean of 51 mg and 20 mg of GR and NeoGB) for 5 weeks, with a subsequent follow-up phase of the same time (Table 2) [66]. On the other hand, the mean basal concentrations of SFN-NAC, the main compound detected, increased by almost 37 and 60-fold after 5 weeks of broccoli sprouts consumption for men and postmenopausal women, respectively, without significant differences between either of the two experimental groups. Similarly, SFN-CYS increased as a result of the dietary intervention by almost 28-fold relative to the basal conditions. The sum of SFN forms and conjugates (SFN-NAC and SFN-CYS) after 5 weeks of intervention was 2.68 nmol/mg of creatinine for premenopausal women, 6.78 nmol/mg of creatinine for postmenopausal women, and 2.43 nmol/mg creatinine for men, thus, postmenopausal women appeared to metabolize ITC to a greater extent [66]. It is known that genetic polymorphisms in the *GST M1/T1/P1* and *NAT* genes encoding the metabolic enzymes glutathione-*S*- and *N*-acetyl-transferases are crucial inter-individual variability factors [37]. According to these results, the authors suggested that SFN-NAC, SFN-CYS, and SFN can be considered suitable biomarkers of Brassica intake [51]. The 20-day wash-out period was enough for the clearance of all metabolites derived from the ingestion of broccoli sprouts. The use of recipes that did not affect freshness contributed to the non-inactivation of the myrosinase enzyme. Therefore, from the intervention studies summarized here, it can be concluded that the daily consumption of broccoli sprouts provides bioavailable and biologically active ITC likely to exert protective effects against inflammation [72].

Aside from ITC, epithionitriles are the main hydrolysis products of GSL. They are formed enzymatically by the action of the epithiospecific protein (ESP) and are characterized by a terminal double bond. Their precursors can be the GSL sinigrin, as well as gluconapine, glucobrassicanapine, progoitrin, epiprogoitrin, and gluconapoliiferin [9,73]. In an *n-of-one* longitudinal study designed to set up conditions of analysis of these compounds, one subject ingested 5 g of sprouts of white cabbage (*Brassica oleracea* var. *capitata* f. *alba* cv. Jetma RZ F1) for 8 days. Specific urine analyses were performed to identify the epithionitrile metabolite 1-cyano-2,3-epithiopropane (CETP), the most important epithionitrile in the human diet (Table 2). Further, an additional pilot study was carried out with a very small experimental group (*n* = 3), who were given a shake preparation containing 7.6 g of *B. carinata* sprouts [58]. Small amounts of 1-cyano-3,4-epithiobutane (CETB), 1-cyano-2-hydroxy-3,4-epithionitrile (CHETB), and allyl ITC were detected, as well as allyl-ITC-NAC and an *N*-acetyl-S-(3-cyano-2-(methylsulfonyl)propylcysteine. The highest concentrations of CETP metabolites were found after 3 h of consumption, after which the amounts recorded decreased to reach the pre-intervention levels after 24 h [67]. This study reported that epithionitriles were released due to the high abundance of alkenyl GSL in the presence of ESP. The *N*-acetyl-*S*-(3-cyano-2 methylsulfonyl)propyl-cysteine was detected, suggesting a rapid absorption and metabolism of CETP, as previously described [74]. Therefore, the bioavailability of CETP appears to be as quick as that of ITC [72]. These preliminary studies suggest that epithionitriles are rapidly metabolized, but more research on their bioavailability and identification of potential intermediate metabolites is needed to accurately assess their pharmacokinetics [67].

The utility and limitations of 2-thiothiazolidine-4-carboxylic acid (TTCA) as a marker for monitoring quantitative modifications of bioactive metabolites after ingestion of cruciferous plants in clinical studies have been investigated. A clinical trial including 50 participants who consumed a drink rich in GR or SFN was conducted [53]. The experimental design included a first period of 7 days, during which volunteers consumed a drink rich in GR (in the evening). Afterwards, a 5-day washout period was implemented, followed by a second 7-day drinking period (beverage rich in SFN). The authors hypothesized that this assay would allow the evaluation of the bioavailability of SFN and its mercapturic derivatives by measuring TTCA in urine as a common isothiocyanate-derived metabolite, with the potential to be used as a biomarker of the intake of cruciferous vegetables (Table 2) [68]. As the main conclusion of this work, TTCA, which seems to be produced by all crucifers, regardless of the identity of the GSL/ITC precursor, provided the opportunity to quantify the intake and absorption of organosulfur compounds from any cruciferous vegetable. Indeed, the authors observed increases of up to 60% in the urine concentration of TTCA 12 h after the ingestion of GR and SFN beverages [68]. In addition, further measurements showed that much of the TTCA detected in the human urine was formed in the broccoli beverages before consumption, although in beverages with a high SFN content, some additional TTCA was formed as a result of SFN biotransformation. In this regard, it is essential to highlight that the highest levels of TTCA were found in broccoli sprouts as compared with the mature plant and other crucifers, due to a higher concentration of GR per dry weight, producing higher amounts of SFN [75]. These results support evidence reported in a previous study that described TTCA as a potential biomarker of cruciferous vegetable intake in clinical trials and nutritional epidemiological studies [76]. However, from a biological point of view, it is important to remark that unlike SFN, TTCA does not activate Nrf2-mediated cytoprotective signaling [68].

Optimizing the bioavailability of bioactive compounds in the diet is as important as maintaining a high food content. This fact promoted several investigations on the extent to which food modifications may influence the absolute bioavailability of bioactive compounds. In this regard, an exploratory study by Oliviero et al., using freeze-dried broccoli sprout powder incorporated into protein gels (gelatin), fibers (alginate), and lipids (candelilla wax), explored their effect on the bioavailability of ITC. The use of gels incorporating the modulatory agents was considered as a strategy to minimize the modulatory effect of the food structure while providing protection to food components against digestion, increasing the amount of GR and glucoiberin that reached the colon. This is especially relevant, given the enzymatic capacity of the microbiota to convert GSL into bioactive ITC. An intervention study included five participants and analyzed the bioavailability of ITC, by measuring the 24-urine concentration of SFN and iberin (IB), the cumulative excretion, bioavailability, and time of maximum excretion peak [24]. The novelty of this study lies in the treatment of the powdered formula with myrosinase to force the conversion of GR into SFN, which was analyzed in comparison with controls containing inactive myrosinase. The main results evidenced very similar excretion patterns for SFN and IB. In addition, it was observed that the excretion of SFN and IB was delayed by 6–7 h in volunteers consuming the control sources of GR and GI containing inactivated myrosinase, as compared with the formulas including the active form of the enzyme. The shape of the curves and the time of peak excretion (Tmax) of SFN and iberin were very similar, suggesting matching pharmacokinetics for the two ITC. This result was justified by the authors on the structural similarity between SFN and iberin, which differ only in one methyl group on the side chain, according to Pilipzzuk et al. [77]. Additionally, in this work, the bioavailability was calculated on the basis that the cumulative excretion was 3-fold higher when ingesting formulations with active myrosinase for both SFN and IB. These results confirmed the previously reported fast absorption of ITC formed in the food matrix before ingestion, as well as that of ITC produced from GSL during chewing of the dietary source in the presence of active myrosinase. Nonetheless, the high dispersion of results due to inter-individual variability, primarily attributed to differences in microbiota population and the absorption yield in the small intestine, as well as the interconversion between SFN and erucin, did not allow extracting further conclusions on bioavailability [24]. In light of the lack of significant differences between volunteers who consumed broccoli sprout powder incorporated into protein gels, the authors noticed the importance of standardizing the rehydration procedure to prepare the gels to obtain consistent and comparable data. The reduction of the bioavailability of ITC by fiber, also observed by Oliviero et al. [19,60], may be explained by the binding of these compounds to the hydroxyl groups of alginate or by physical entrapment in the gel network, as described by Corstens et al., on the use of emulsion-alginate beads on food formulation [78]. All of this can be considered preliminary for future research on the effects of the structure and composition of food matrices [24].

To finish with the clinical cases using sprouts as sources of GSL, a study was carried out using BSE containing SFN in a gel capsule to study its role as a chemopreventive agent, through inhibition of ultraviolet radiation-induced damage and tumor progression in the skin. The evaluation was performed by administrating three doses (50, 100, or 200 μmol, randomly) of oral BSE-SFN for 28 days, to 17 at-risk patients (5 men and 12 women, within the range of age 22–66 years, with two or more atypical nevi and a history of melanoma). A visual control was maintained by taking images of the atypical nevi and analyzing plasma samples and SFN levels in the skin, demonstrating a dose–response relationship. Statistically significant decreases in the plasma level of proinflammatory cytokines and an increase in tumor suppressor decorin were found, from day 1 to 28, thus providing preliminary evidence of the biochemical activity of SFN by oral ingestion on cutaneous nevi [70].

Regarding the proliferation marker Ki-67, it was expressed at the beginning of the study and on day 28 in keratinocytes, but only rarely in nevi melanocytes. However, the anti-apoptotic marker (Bcl-2) was more prominently expressed. Some nevi decreased in size, and generally, the increase in size was less pronounced in the groups receiving the higher dose of BSE-SFN, showing an apparent tendency to reduce the nevi growth, with the most notable effect being observed in the 200 μmol dose group [70]. Lower concentrations of SFN in skin as compared to those in plasma, may reflect relatively poor skin perfusion as compared to other organs [79,80], which implies that increased doses or dose frequency may be required to achieve more sustained levels in tissues [81]. The results of this pilot study support the potential biological impact, needing a larger study of oral BSE-SFN at 200 μmol per day for a longer treatment period of several months, or considering higher or more frequent doses of BSE-SFN [70].

## 5. Evidence of Dietary Interventions Using Cruciferous Vegetables and Derived Food Products

For the evaluation of the impact of consuming cruciferous foods (and derived products), most research is based on interventions and epidemiological studies using the marketable or commercial parts of adult plants (e.g., inflorescences or heads, roots, leaves, etc.) (Table 3). Frequently, broccoli is consumed in the cooked form due to the organoleptic acceptance of this processing alternative by consumers. However, the limited myrosinase activity that remains after boiling has to be counteracted to take advantage of the biological benefits of ITC. In this regard, the combination of dietary sources of GSL (e.g., cooked broccoli) with a source of active myrosinase (e.g., daikon radish) has been frequently explored to improve the bioavailability of these compounds. Charron et al. (2018) developed a nutritional trial including overweight/obese adults—body mass index (BMI) > 25 kg/m^2^—(HiBMI) as the target population, who consumed boiled broccoli and raw daikon radish (active myrosinase) for 17 days [82]. This study, which included 18 volunteers, sought to uncover whether daikon radish myrosinase affected the absorption and metabolism of ITC derived from broccoli GSL and their mercapturic derivatives, as well as to discover the relationships of such traits with BMI, the genotype of glutathione-*S*-transferase µ 1 (*GSTM1*), and/or sex. The experimental groups were composed by volunteers who consumed (1) a control diet without broccoli for 16 days (no broccoli (NB)—control group) or (2) the control diet supplemented with 200 g of cooked broccoli and 20 g of raw daikon radish (source of myrosinase) daily for 15 days, and 100 g of broccoli and 10 g of daikon radish on day 16 (dietary broccoli (DB)—experimental group). Both NB and DB experimental groups consumed 200 g of broccoli and 20 g of daikon radish for 17 days (Table 3). The occurrence of GSL and ITC derivatives was monitored in plasma and 24-h urine. The absorption of GSL was observed using the production of metabolic derivatives in plasma and urine as indicators, which allowed the comparison of overweight volunteers (BMI > 25 kg/m^2^) with healthy-weight subjects (BMI < 24 kg/m^2^; LoBMI) [82]. Interestingly, the different diets did not result in significant modifications of the plasma and urine concentrations of GSL, ITC, and the mercapturic derivatives. Although GR made up 94% of the GSL supplied, SFN and its derived metabolites were 32% and 34% of total plasma AUC for the NB and DB diets, respectively, and 53% and 54% of the urinary metabolites for the NB and DB diets, correspondingly. The total metabolites measured in plasma were the glutathione, cysteine-glycine, cysteine, and *N*-acetylcysteine derivatives of SFN and ER, with ER-CysGly being prominent in plasma. On the contrary, in urine, ER-NAC and SFN-NAC were the predominant metabolites [82]. From these results, it was suggested that the previous exposure to SFN could increase the glutathione *S*-transferase activity in enterocytes, thus improving the metabolism and secretion of metabolites to the intestine as observed in vivo [83]. However, the relationship between this mechanism and BMI was not established.

Interestingly, in this study, no association of the *GSTM1* genotype or sex could be established with the bioavailability of ITC and mercapturic derivatives [82]. The authors speculated on the interactive effect of daily broccoli consumption by people with a BMI > 26 kg/m^2^ on the plasma levels of SFN and mercapturic derivatives produced, suggesting that broccoli consumption may have a mechanistic interest by providing the capacity to modulate disease risk tentatively, due to the interactions with the intestinal microbiota. Indeed, these two factors (BMI and microbiota) could be interconnected, as the gut microbiome varies with BMI [84,85]. This is of special importance, as the microbial capacity to metabolize SFN is strongly linked with specific bacterial strains and their metabolic capacity [82]. However, as recognized by the authors themselves, this work was affected by a serious concern related to the different fiber content of the diets in both control and broccoli-consuming experimental groups, which is of critical importance, as it is a central factor that affects the absorption of food components [82]. Aside from this, dietary fiber is strongly associated with the intestinal microbiota profile, as it modifies the intestinal transit time. Once again, the consideration of the microbiota and the microbiome in the context of interactions with the bioactive compounds, and their delivery to target organs, is becoming a key factor in research with food bioactives for health outcomes [31,65].

Charron et al., in a subsequent study using cooked broccoli without supplementary plant myrosinase, explored how the daily consumption affected the metabolism of GSL into ITC and downstream metabolites [86]. Upon an intervention with cooked broccoli without additional myrosinase, it was concluded that the predominant metabolite in plasma was erucin-cysteine-glycine (ERN-CysGly, 50% of the total), followed by SFN-CysGly and unesterified SFN (14% and 13%, correspondingly), erucin-cysteine (ERN-Cys, 7%), SFN-GSH (6%), SFN-NAC (5%), SFN-Cys (2%), erucin-*N*-acetylcysteine (ERN-NAC, 2%), and erucin-glutathione (ERN-GSH, <1%). In urine, the predominant metabolites were ERN-NAC (39%) and SFN-NAC (38%), followed by SFN-Cys (11%), ERN-Cys (7%), and SFN (4%). Of the 151.2 μmol of GR and GE provided, 10.8% and 12.0% were recovered as metabolites excreted in the 24-h urine for the NB and DB diets, respectively. The most relevant results obtained showed that although there was no direct diet effect, a BMI effect was again significant, confirming the main conclusions of the previous study. Ultimately, 8.5% of GR and GE-derived metabolites were recovered in the urine of subjects with LoBMI, while in HiBMI patients, the recovery rate was significantly higher at 15.1% [86]. These results evidence a pronounced effect of BMI on the absorption and metabolism of GSL, regardless of the frequency of broccoli consumption. Therefore, the influence of BMI on the metabolism and absorption of GSL should be a factor to consider in clinical trials that evaluate the real impact of these compounds on health. These findings should be analyzed in light of previous research by other authors. Indeed, Conaway et al. reported that the consumption of cooked broccoli resulted in a decrease in the accumulation of urinary metabolites of GSL in 24 h as compared to fresh broccoli [87]. The plasma response curves for the LoBMI and HiBMI groups were similar for approximately 0 to 5 h and then diverged, with plasma levels increasing at a higher rate in people with a high BMI as compared to those with a lower BMI. This difference in plasma levels could indicate a difference in the response of the colon and microbiota associated with the metabolic pathways that affect GSL [88]. This highlights the importance of the BMI factor with a positive incidence for overweight patients, with differences in urinary excretion of derived metabolites between LoBMI and HiBMI, with an excretion higher by 6.6% for the latter. Differences in intestinal transit time may play a role. All of this warrants further investigations to provide a more effective dietary guide for optimal health [86].

**Table 3:** Dietary interventions using mature cruciferous vegetables and derived products.

Dietary Source-Intervention Time	Sulfur-nitrogen-Based Compounds-Dose	Biological Samples	Subjects’ Characteristics	Study Record Identifier (NCT Number)	Metabolites and Conjugates–Concentration Range	Analytical Technique	Reference
Commercially frozen broccoli 17 days once a day	GR and Glucoerucin 200 g of broccoli (providing 97.5 µmol of glucoraphanin and 5.8 µmol of glucoerucin)	Plasma and urine	Healthy subjects: 10 women and 8 men, 37–65 years of age	NCT02346812	SFN	UHPLC-QqQ-MS/MS	[82]
					SFN-GSH		
					SFN-CysGly		
					SFN-Cys		
					SFN-NAC *		
					Erucin-GSH		
					Erucin-CysGly > 37%		
					Erucin-Cys		
					Erucin-NAC * * (The two compounds represent > 41%)		
					SFN + sulforaphane metabolites		
Blanched and frozen broccoli 26 days	GR and glucoerucin 200 g (the day before the study treated group eat only 100 g and on the day of the study all volunteers treated and non-treated eat 200 g)	Plasma and urine	Healthy women and men (between 40 and 70 years old)	NCT03013465	SFN 13%/4% (% plasma/urine)	LC-MS	[86]
					SFN-GSH 6%		
					SFN-CysGly 14%		
					SFN-Cys 2%/11%		
					SFN-NAC 5%/38%		
					Erucin-GSH < 1%		
					Erucin-CysGly 50%		
					Erucin-Cys 7%/7%		
					Erucin-NAC 2%/39%		
Kale and daikon radish 1 day	GSL 250 g of baby kale (steamed weight 263 g), 25 g of uncooked daikon radish	Urine	Healthy adults (32–71 years old)	NCT03449849	I3C: 6 arbitrary units/hour	UHPLC- HRAM-MS	[89]
					MI3C: 1.2 arbitrary units/hour		
					I3-CAL: 3.5 arbitrary units/hour		
					MI3-CAL: 15 arbitrary units/hour		
					I3-CA: 22 arbitrary units/hour		
					MI3-CA: 15 arbitrary units/hour		
					AITC-Cys: 0.5 arbitrary units/hour		
					AITC-NAC: 3 arbitrary units/hour		
					4-methylsulfinyl-3-butenyl isothiocyanate: 23 arbitrary unit/hour		
					4-methylsulfinyl-3-butenyl isothiocyanate-cysteine: 7 arbitrary unit/hour		
					4-methylsulfinyl-3-butenyl isothiocyanate-*N*-acetyl cysteine: 23 arbitrary units/hour		
					Ascorbigen: 2.5 arbitrary unit/hour		
					HABG: 5 arbitrary units/hour		
					MABG: 0.7 arbitrary units/hour		
Raw broccoli 12 day	GR 200 g of uncooked broccoli florets	Plasma and urine	Healthy adults (28–67 years old)	NCT03287115	I3C: 2200 (nmol/mmol cretinine)	UHPLC- HRAM MS	[90]
					I3-CAL: 80		
					I3-CA: 50		
					ABG: 8000		
					SFN: 800		
					SFN-GSH: 5.5		
					SFN-Cys: 150		
					SFN-NAC: 700		
					MI3C: 4500		
					MI3-CAL600		
					4-methylsulfinyl-3-butenyl isothiocyanate 6500		
					MABG 10,000		
					HABG 1000		
Cooked broccoli 1 day	GR 200 g 1 g powdered brown mustard (*Brassica* *juncea*)	Urine	12 Healthy adults between 18 and 64 years	N.A.	SFN-NAC 44.7 ± 33.9 μmol SFN-NAC per gram creatinine (9.8 ± 5.1 µmol SF-NAC per gram creatinine within 24 h without mustard)	HPLC-UV	[90]
Cooked *B. carinata* leaves (ethiopian kale) 4 days	Sinigrin 269 µmol sinigrin per serving (15 g)	Urine	22 Participants (5 males and 17 females), aged 22.7 ± 2.4 years	DRKS00010836	AITC-NAC 9.36 ± 9.81 (24 h after 4 days intake) mol/L urine	LC-ESI-MS/MS	[91]
Raw *B. carinata* leaves 5 days	AITC 177 µmol of AITC per serving (15 g)	Plasma and urine	22 Participants (5 males and 17 females), aged 22.7 ± 2.4 years	DRKS00010836	AITC-NAC 38.07 ± 21.00 (24 h after 4 days intake) mol/L urine	LC-ESI-MS/MS	[91]
					AITC-GSH 53.90 ± 10.17 (2 h after day-5 intake) nmol/L plasma		
					AITC-CysGly 233.07 ± 167.55 (2 h after day-5 intake) nmol/L plasma		
					AITC-Cys 92.71 ± 71.811 (2 h after day-5 intake) nmol/L plasma		
					AITC-NAC 23.32 ± 10.21 (2 h after day-5 intake) nmol/L plasma		

Abbreviations: ABG: ascorbigen; AITC-Cys: allyl isothiocyanate cysteine; AITC-CysGly: allyl isothiocyanate cysteinyl glycine; AITC-GSH: allyl isothiocyanate glutathione; AITC-NAC: allyl isothiocyanate *N*-acetylcysteine; erucin-Cys: erucin cysteine; erucin-CysGly: erucin cysteinyl glycine; erucin-GSH: erucin glutathione; erucin-NAC: erucin *N*-acetylcysteine; GSL: glucosinolate; GR: glucoraphanin; I3 C: indole 3 carbinol; I3-CA: indole 3-carboxilic acid; I3-CAL: indole 3-carboxialdehyde; ITC: isothiocyanates; HABG: hidroxy ascorbigen; MABG: methoxy ascorbigen; MI3C: methoxy indole 3 carbinol; MI3-CA: methoxy indole 3-carboxilic acid; MI3-CAL: methoxy indole 3-carboxialdehyde; N.A.: not available; SFN: sulforaphane; SFN-Cys: sulforaphane cysteine; SFN-CysGly: sulforaphane cysteinyl glycine; SFN-GSH: sulforaphane glutathione; SFN-NAC: sulforaphane *N*-acetylcysteine. NCT Number or record study number assigned in ClinicalTrials.gov.

The combination of targeted and untargeted metabolomics approaches may allow for obtaining comprehensive metabolic profiles within the frame of human dietary intervention studies. In a metabolomics study, urine samples from healthy volunteers, after eating a single breakfast meal of kale (250 g) and raw daikon radish (25 g), were analyzed to determine and quantify the major GSL metabolites (Table 3). The urinary metabolome was investigated in different periods after intake, finding up to 14 different GSL derivatives, among which 4-methylsulfinyl-3-butenyl ITC (MESBT), 4-methylsulfinyl-3-butenyl ITC-Cys, and 4-methylsulfinyl-3-butenyl-GSL-NAC were observed for the first time in human urine. The major GSL in kale and daikon radish were identified and quantified as desulfo-GSL, with a total content of 2.97 and 5.65 μmol/g, respectively. The application of specific analytical methods for metabolites of interest, according to the available knowledge on the GSL composition of kale and daikon radish, allows gaining further insights into the inference of these organosulfur compounds with potential metabolites based on the metabolic pathways currently described, and also allows for the development of a list of metabolites on which to place our interest. The main GSL in kale were identified as sinigrin (allyl-GSL) and glucobrassicin (3-indolylmethyl-GSL, GBS), while for daikon radish, the predominance of glucoraphasatin (4-methylthio-3butenyl-GSL), glucoraphenin (4-methylsulfinyl-3- butenyl-GSL), and 1-methoxy-GSL was described. The ITC metabolites derived from sinigrin, glucoraphenin, and glucoraphasatin were predicted to be allyl-ITC (AITC), MESBT, and 4-methylthio-3-butenyl ITC (METBT), as well as their mercapturic derivatives [89]. Interestingly, Nakamura et al. reported a correlation between the potency of antimutagenicity and the amount of METBT derived from daikon radish [92]. Note that the metabolites of kale indole GSL, such as GBS and methoxyglucobrassicin (MeGB), are very similar to the metabolites detected after broccoli consumption, which are indole and methoxylated-indole derivatives. Ascorbigen (ABG), hydroxy ascorbigen (HABG), and methoxy ascorbigen (MABG) were also detected, which is consistent with a previous broccoli feeding study [93]. The potential human health benefits of MESBT thus merit further attention because of its similarities with SFN [89].

Untargeted and targeted metabolomic approaches were used to evaluate the physiological responses after broccoli consumption within the frame of a pilot dietary intervention in which volunteers (*n* = 6) ingested 200 g of raw broccoli florets. The metabolomic analysis revealed modifications in the presence of 13 GSL metabolites in urine, including free SFN, SFN-GSH, SFN-Cys, and SFN-NAC from GR, as well as I3C, indole-3-carboxaldehyde (I3-CAL), and indole-3-carboxylic acid (I3-CA) from indolic GSL (GB and relatives), and of eight metabolites in plasma (SFN, SFN-Cys and SFN-NAC, and I3-CA, I3-CAL, and ABG and MABG (Table 3). Additionally, the presence of methoxyl-indole GSL GBS, NeoGB, and MeGB was observed. The total GSL concentration was 14.67 µmol/g dry weight of the meal. After dietary intake, the inter-individual variability was very high in terms of the profiles and urine concentration of GSL metabolites. Consequently, by comparing volunteers, the authors stressed the different specific metabolite profiles as informative [93]. In this regard, it should be noted that the urinary GB metabolites, specifically I3-CA and I3-CAL, should not be considered appropriate markers of the dietary intake of broccoli, as they also occur endogenously in humans [94], even though their concentration increased significantly after broccoli consumption [93]. The metabolites of methoxyl GSL identified in urine were methoxyl-indole-3-carbinol (MI3C), methoxyl-indole-3-carboxaldehyde (MI3CAL), methoxyl-indole-3-carboxylic acid (MI3CA), and MABG. Additionally, hydroxyl-ABGs were found. However, regrettably, the absence of standard references did not allow differentiating the metabolites arising from isomeric 1-methoxy- or 4-methoxy-glucobrassicin, for instance. The metabolites of MGB detected in plasma were MI3CA and MABG. Compared to urine concentrations, the plasma metabolite concentrations were much lower. These results suggest that SFN, SFN-Cys, and SFN-NAC (metabolites predominating in plasma after broccoli consumption), indole ascorbygen, and methoxyl ascorbygen may be further considered as candidate markers for Brassica vegetable intake [93].

Another study aimed to investigate the bioavailability of SFN, by measuring the production of the SFN-NAC in urine after the consumption of cooked broccoli (200 g) supplemented with brown mustard seed powder (*Brassica juncea* L Czern, 1 g) vs. the intake of broccoli without an external source of myrosinase, in 12 healthy adults (Table 3). After 8 min of cooking at 100 °C, no myrosinase activity was recorded. The SFN content in pure unprocessed broccoli was 2.05 μmol/g dry weight (dw), which was reduced down to 1.06 μmol/g dw after vacuum cooking [90]. As it is known, broccoli processing at low temperatures (less than 50 °C) is more suitable, as it promotes SFN formation [95], as the epithiospecific protein activity is prominent at this temperature [96]. However, the same authors suggested that a cooking temperature of up to 60 °C allows the formation of SFN. In addition, it should be noted that when brown mustard was added to the cooked broccoli sample (in vitro), there was a significant eight-fold increase in SFN content (8.58 μmol/g dw), thus improving the conversion of the intact GR into its bioactive counterpart (SFN). This conversion has been described as a result of the enzymatic activity of the myrosinase from mustard, which is more robust and thermally stable than the broccoli isoform [97,98]. Other strategies can also enhance myrosinase activity. For example, pre-soaking broccoli florets in water at 37 °C for 90 min promotes hydrolysis before the enzyme denatures upon cooking, leading to an increase of the SFN concentration up to 2.8-fold [99]. After consumption of cooked broccoli alone, subjects excreted a mean of 9.8 μmol SFN-NAC per gram of creatinine in 24-h urine, whereas after ingestion of cooked broccoli with brown mustard powder, they excreted 44.7 μmol of SFN-NAC per gram of creatinine in 24-h urine, demonstrating that mustard powder myrosinase displays a more efficient hydrolysis activity, transforming GR into SFN more efficiently. For the cooked broccoli group, the amount of SFN should be related to the conversion of GSL by the intestinal microbiota, but with lower yields [69,80]. The rate of increase when mustard was added to cooked broccoli in vitro was greater than that observed in the in vivo study. Therefore, it may be concluded that adding mustard greatly improves the formation of SFN, enhancing its concentration by almost eight times, as well as its bioavailability and metabolism, increasing the formation of SFN-NAC by more than four times [90].

Shifting to other cruciferous food, the leaves and seeds of Ethiopian kale (*Brassica carinata*) are very rich in sinigrin and, therefore, represent a relevant source of the chemopreventive sinigrin-derived ITC, AITC [100,101]. In the frame of recent in vitro research, new evidence of the protective effects of AITC against liver carcinogens, such as the mycotoxin aflatoxin B1 (AFB1) promoter, has been retrieved [69,80]. In this study, 22 healthy young subjects consumed cooked or raw leaves of Ethiopian kale for five days (Table 3). The ITC were produced during cooking, as the raw leaves contained 177 µmol AITC per serving, with 6 µmol residual sinigrin on average, while the cooked leaves contained 269 µmol sinigrin and no AITC recovered in plasma or urine, except for AITC-NAC, which was found only in low concentrations in urine. Different mercapturic derivatives of AITC were detected in the plasma of subjects consuming the raw leaf preparation, and a four-fold higher amount of AITC-NAC was detected in the urine sample. With respect to the functional implication associated with bioavailable AITC, the DNA damage of peripheral blood mononuclear cells (PBMC) induced by AFB1 ex vivo was analyzed, and a significant reduction of DNA damage was observed when consuming cooked leaves. This finding indicates that AITC does not primarily mediate the antigenotoxic effect, and tentatively, additional bioactive compounds present in the food matrix considered (e.g., (poly)phenols or vitamins) could participate in the activity [102]. Concerning the specific functionalities attributed to ITC, a decrease in inflammatory mediators has been described for ITC in general, and AITC in particular, through inhibition of the nuclear factor NF-κB and blocking of the COX-2 (cyclooxygenase-2) signaling pathway [103,104]. The mediation of the natural prostaglandin E_2_ (PGE_2_), a metabolite produced by COX-2, contributes to inflammation [105], which is involved in the regulation of the immune response. In this trial, plasma PGE_2_ levels were significantly lower with the AITC-containing preparation. Therefore, this dietary intervention seems important for enhancing the anti-inflammatory capacity, but the reported results support the use of these bioactives to counteract the exposure to aflatoxins [91].

## 6. Other Dietary Sources of Glucosinolates: Seeds, Extracts, or Formulas Enriched in GSL

A range of studies have focused on using extracts rich in bioactive compounds from cruciferous foods, formulated as ingredients, to overcome the rising trend of reducing the intake of fresh or cooked Brassica foods or, beyond this, looking into the possibility of obtaining improved new foods, ingredients, or supplements with higher concentrations of the target bioactive compounds (e.g., pill or capsule). The advance towards the consecution of these new formulas would facilitate the follow-up of patients, volunteers, and the control of the dose, in good agreement with the request from the clinical advisers, or because of the characteristics of the patients (e.g., patients or volunteers not familiar with the consumption of Brassica foods) (Table 4). Indeed, the challenge is to combine a high dosage of potentially-effective compounds, with a reduced chance of participant drop-off, in a more pharmacology-like study or intervention. One example of this new trend is BroccoMax^®^ (Jarrow Formulas^®^, Los Angeles, CA, USA), an encapsulated broccoli seed extract (source of SFN in a deoiled formulation to avoid antinutrients such as erucic acid—according to claims on the label) [25]. These dietary supplements are useful for dietary interventions facilitating a controlled daily dose in the administration.

In a comparative study between non-pregnant and preeclamptic women, the bioavailability of SFN and its conjugates (SFN-GSH, SFN-CysGly, SFN-Cys, and SFN-NAC) were studied as a possible adjuvant therapy by using a formulation with active (BroccoMax^®^, Jarrow Formulas®, Los Angeles, CA, USA) or inactive (Broccoli Sprout Extract^TM^, Source Naturals^®^, Scotts Valey, CA, USA) myrosinase in non-pregnant women, as well as the effects of the intake of BroccoMax^®^ on blood pressure in preeclamptic volunteers [30]. A combined profile of all SFN metabolites in plasma samples evidenced that the activated formulation provided higher levels of total metabolites, with an area under the curve (AUC) and a mean maximum concentration (C_max_) higher than those obtained when administering extracts with inactivated myrosinase. Thus, this study confirmed the enhanced benefits associated with the presence of the enzyme responsible for hydrolyzing GSL after consumption. Reasonable levels of metabolites were also obtained after consumption of the non-activated preparation, showing that bacterial hydrolysis may contribute more to GSL transformation than expected [30]. When total exposure was compared, the AUC in non-pregnant women was approximately double. There was a trend towards a non-significant reduction (~10%, using the biomarker sFlt-1) in diastolic blood pressure, regardless of the dose. SFN may improve endothelial-dependent vasodilator effects and blood pressure, the former perhaps through antioxidant pathways and relaxation of peripheral vascular smooth muscle. Therefore, any decrease in sFlt-1 could result in a reduction in placental oxidative stress and an improvement in mitochondrial trophoblast function. Pharmacokinetic studies suggest that multiple daily doses are required for maintaining levels with therapeutic action through bioavailable ITC metabolites in plasma. Taking into account changes in drug distribution, metabolism, and elimination during pregnancy, the levels of SFN and its derived metabolites in women with preeclampsia may require up to twice the recommended dose of BroccoMax^®^ to be effective [30].

**Table 4:** Seeds, Extracts, or formulas enriched in glucosinolates.

Dietary Source- Intervention Time	Sulfur-Nitrogen-Based Compounds-Dose	Biological Samples	Subjects Characteristics	Study Record Identifier (NCT Number)	Metabolites and Conjugates-Concentration Range		Analytical Technique	Reference
Broccoli seed extract (BroccoMax^®^) and broccoli sprout extract 1 day	GR 32/64 mg sulforaphane	Plasma	Healthy women aged 18–35 years	N.A.	SFN: 125/150 nM (non-active/active)		LC-MS	[30]
					SFN-GSH: 140/280 nM			
					SFN-CysGly: 300/550 nM			
					SFN-Cys: 100/160 nM			
					SFN-NAC: 48/66 nM			
Broccoli seed extract (BroccoMax^®^) 1 day	GR 32/64 mg sulforaphane	Plasma	Women with a singleton pregnancy and a diagnosis of preeclampsia or gestational hypertension, >18 years old	N.A.	SFN: 44/80 nM		LC-MS	[30]
					SFN-GSH: 60/160 nM			
					SFN-CysGly: 110/180			
					SFN-Cys: 50/60 nM			
					SFN-NAC: 60/120 nM			
Broccoli seed extract (BroccoMax^®^) 1 day	GR 32/64 mg sulforaphane	Plasma	Healthy nulliparous women aged between 20 and 23 years	N.A.	SFN: 183.5/206.5 nM		LC-MS	[106]
					SFN-GSH: 150.1/240.8 nM			
					SFN-CysGly: 408/419.2 nM			
					SFN-Cys 113.8/112.2 nM			
					SFN-NAC: 74.3/35.6 nM			
Broccoli powder in soup and mustard seeds 1 day	GSL 200 mL soup	Ileal fluid	Ileostomates 53.3 ± 9.2 years	NCT04113928	SFN: 1.05 µM		HPLC–UV/GC–MS	[107]
					Glucoiberin: 4–22 µM			
					Sinigrin: 0 µM			
					Gluconapin: 6–46 µM			
					Glucoerucin: 0–32 µM			
					GB: 2–23 µM			
					Gluconasturtiin: 0–3 µM			
					GR: 30–60 µM			
					Glucoalysin: 1–4 µM			
					HGB: 0–2 µM			
					NeoGB: 3–24 µM			
					MGB: 4–48 µM			
Broccoli seed and sprout extract supplement Avmacol^®^ 1 day	GR 8 tablets per day per subject, estimated to deliver about 369 μmol/subject/day of GR	Urine	Healthy adults (24–69 years old)	N.A.	25.67% (uncoated and no omeprazole); 35.48% (coated and no omeprazole); 33.59% (uncoated and omeprazole); 36.41% (coated and omeprazole) conversion efficiency		cyclocondensation reaction-HPLC assay	[108]
Broccoli seed and sprout extract supplement Avmacol^®^ 15 weeks	GR 2.2 μmol/kg/day	Plasma	Children 3–12 with autism spectrum disorder	NCT02561481	Dithiocarbamates: SFN group week 0: 0.007; SFN group week 7: 0.299; SFN group week 15: 0.329 nmol/ml		Cyclocondensation reaction-HPLC assay	[109]
Broccoli soup and broccoli soup with mustard 1 day	GSL 200 ml	Ileal fluid	Ileostomy subjects	NCT04113928	Kynurenine: 99.5 (without mustard seeds) and 42.8 (with mustard seeds) ng		UHPLC-QqQ	[110]
					Tryptamine: Ileal fluid content: 11.7 (without mustard seeds) and 13.2 (with mustard seeds) ng			
					Indole-3-lactic acid: Ileal fluid content: 88.6 (without mustard seeds) and 308.8 (with mustard seeds) ng			
					Indole-3-aldehyde: Ileal fluid content: 34.4 (without mustard seeds) and 103.9 (with mustard seeds) ng			
					Indole-3-acetic acid: Ileal fluid content: 18.2 (without mustard seeds) and 28.0 (with mustard seeds) ng			
*Nasturtium* leaves suspension made from freeze-dried leaves 48 h	Benzyl glucosinolate (Glucotropaeolin) 1.71 µmol of Benzyl-GSL and 191 µmol of BITC	Plasma and urine	Four healthy women aged between 26 and 61	N.A.	BITC-GSH:-(in urine)	Total metabolites in plasma: 0.36–1.06 in plama µmol/L	LC–ESI–MS/MS/GC-MS/MS	[111]
					BITC-CysGly: -			
					BITC-Cys: 1–2 µmol/L, maximum after 4 h			
					BITC-NAC: 60 µmol/L maximum after 4 h consumption			
Bread enriched with *nasturtium* leaves 48 h	4.3 µmol of Benzyl-GSL and 2.48 µmol of BITC	Plasma and urine	Three healthy women aged between 26 and 61	N.A.	BITC-GSH: -	Total metabolites in plasma: 0.24–0.35 µmol/L	LC–ESI–MS/MS/GC-MS/MS	[111]
					BITC-CysGly: -			
					BITC-Cys: 0.2–0.5 µmol/L			
					BITC-NAC: 10–20 µmol/L, maximum after 4–6 h			
*Nasturtium* leaves suspension made from freeze-dried leaves 48 h	4.3 µmol of Benzyl-GSL and 2.48 µmol of BITC	Plasma	Healthy women aged between 26 and 61	N.A.	BITC-Lys: <0.2 µmol/L		LC–ESI–MS/MS/GC-MS/MS	[111]
					BITC-Cys: <0.2 µmol/L			
*Nasturtium* leaves suspension made from freeze-dried leaves 48 h	1.71 µmol of Benzyl-GSL and 191 µmol of BITC	Breath	Healthy women aged between 26 and 61	N.A.	BITC: Individual time courses of exhaling both breakdown products among subjects, 0.03–5.89 nmol L^−1^		LC–ESI–MS/MS/GC-MS/MS	[111]
Bread enriched with *nasturtium* leaves 48 h	4.3 µmol of Benzyl-GSL and 2.48 µmol of BITC	Breath	Healthy women aged between 26 and 61	N.A.	BITC: Individual time courses of exhaling both breakdown products among subjects, 0.03–5.89 nmol L^−1^		LC–ESI–MS/MS/GC-MS/MS	[111]
*Nasturtium* leaves suspension made from freeze-dried leaves	1.71 µmol of Benzyl-GSL and 191 µmol of BITC.	Urine	Healthy women aged between 26 and 61	N.A.	BITC: 2.0–8.0 µmol/L		LC–ESI–MS/MS/GC-MS/MS	[111]
Bread enriched with *nasturtium* leaves	4.3 µmol of Benzyl-GSL and 2.48 µmol of BITC	Urine	Healthy women aged between 26 and 61	N.A.	BITC: 2.0–6.0 µmol/L		LC–ESI–MS/MS/GC-MS/MS	[111]
Cooked broccoli, with powdered brown mustard (*Brassica* *juncea*) 1 day	GR 200 g broccoli, 1 g powdered brown mustard (*Brassica* *juncea*)	Urine	12 healthy adults between 18 and 64 years	N.A.	SFN-NAC: 44.7 ± 33.9 µmol SFN-NAC per gram creatinine within 24 h (without mustard: 9.8 ± 5.1 µmol SF-NAC per gram creatinine within 24 h)		HPLC-UV	[90]
Baked snack food containing equivalent phytochemicals1 day	GR 12.7 (glucoraphanin) mg	Urine	Healthy adults (18 females and 10 were premenopausal), average 42 years (age range 20–68 year)	NCT02231502	SFN: 36.25 ± 27.9 nmol/mg intake		HPLC-QTrap	[112]
Microwaved vegetables 1 day	12.6 (glucoraphanin) mg	Urine	Healthy adults (18 females and 10 were premenopausal), average 42 years (age range 20–68 year)	NCT02231502	SFN-NAC: 272.17 ± 280.8 nmol/mg intake		HPLC-QTrap	[112]
Baked snack food containing equivalent phytochemicals1 day	12.7 (glucoraphanin) mg	Urine	Healthy adults (18 females and 10 were premenopausal), average 42 years (age range 20–68 year)	NCT02231502	SFN: 43.72 ± 44.2 nmol/mg intake		HPLC-QTrap	[112]
Microwaved vegetables 1 day	12.6 (glucoraphanin) mg	Urine	Healthy adults (18 females and 10 were premenopausal), average 42 years (age range 20–68 year)	NCT02231502	SFN-NAC: 508.54 ± 450.9 nmol/mg intake		HPLC-QTrap	[112]
Standard broccoli soup 1 day	GSL: 84 ± 2.8 µmoles glucoraphanin per broccoli soup	Plasma	10 participants (3 Men and 7 women) aged 18–65 years	NCT02300324	GR: 0.01 ± 0.01 μmol L^−1^		UPLC–MS/MS	[29]
BENEFORTE broccoli soup 1 day	280 ± 8.8 µmoles glucoraphanin per broccoli soup				GR: 0.03 ± 0.01 μmol L^−1^			
Broccoli soup 1 day	452 ± 10.6 µmoles glucoraphanin per broccoli soup				GR: 0.04 ± 0.02 μmol L^−1^			
Standard broccoli soup 1 day	GR: 84 ± 2.8 µmoles glucoraphanin per broccoli soup	Urine			GR and glucoerucin: 0.54 ± 0.29 μmol/24 h			
BENEFORTE broccoli soup 1 day	280 ± 8.8 µmoles glucoraphanin per broccoli soup				GR and glucoerucin: 1.44 ± 0.66 μmol/24 h			
Broccoli soup 1 day					GR and glucoerucin: 2.12 ± 0.98 μmol/24 h			

Abbreviations: BITC: benzyl isothiocyanate; BITC-Cys: benzyl isothiocyanate cysteine; BITC-CysGly: benzyl isothiocyanate cysteinyl glycine; BITC-GSH: benzyl isothiocyanate glutathione; BITC-NAC: benzyl isothiocyanate *N*-acetylcysteine; GB: glucobrassicin; GSL: glucosinolate; HGB: hydroxy glucobrassicin; MGB: 4-methoxy glucobrassicin; N.A.: not available; NeoGB: neoglucobrassicin; SFN: sulforaphane; SFN-GSH: sulforaphane glutathione; SFN-Cys: sulforaphane cysteine; SFN-CysGly: sulforaphane cysteinyl glycine; SFN-NAC: sulforaphane *N*-acetylcysteine; GR: glucoraphanin. NCT Number or record study number assigned in ClinicalTrials.gov.

When investigating the alternatives to enhance the bioavailability of SFN to take advantage of its biological potential, stabilizing SFN becomes an important issue that needs to be addressed. In this concern, SFN was stabilized with α-cyclodextrin to improve the administration of SFN for clinical use, and the product was given to women aged 18–35 who had never been pregnant [25,86]. Thus, the determination of the appearance of the average peak of metabolites combined with 120 mg of broccoli seed extract (~32 mg SFN), was similar when using 350 mg of pure broccoli seed powder [86]. It is noteworthy that the pharmacokinetics showed the complete excretion 8 h after consumption. However, for the volunteers who ingested SFN in capsules instead of a liquid extract, excretion peaked almost 2 h later [86].

An additional challenge that needs to be addressed is the persistence of broccoli phytochemicals in the upper gastrointestinal tract and their true functionality [113]. This gap in knowledge was investigated by Abukhabta et al. by analyzing the ileal fluid of 11 ileostomy subjects, in a crossover design, with broccoli soup prepared with and without mustard seeds [107]. The broccoli soup contained 26.5 µmol SFN per 200 mL, but after adding 2% mustard seed powder at the cooling stage (~60 °C), the SFN level increased to 102 µmol per 200 mL [107]. This increase was attributed to the isomerase isoform present in more resistant mustard seeds, which, when added to heat-processed broccoli, caused 3 to 5 times more SFN formation (Table 4). The mean SFNe (sulforaphane-enriched extracts) content in the ileal fluids were <1% of the SFNe applied to the soup (indicating early absorption in the small intestine) [83], but the addition of mustard seed (MS) significantly increased the content by six times (augmenting the colonic availability). These results concluded that the addition of low concentrations of seed mustard has the potential to improve the formation of SFN in the intestinal lumen. This is of particular relevance as SFN has been characterized in vitro with regard to the capacity to exert antibacterial effects against enteropathogens (most of them involved in the pathogenesis of intestinal bowel disease) [107,114].

On the other hand, the extracts of cooked broccoli showed considerable antimicrobial activity, while those enriched with mustard seeds exhibited a significantly higher activity, perhaps due to higher levels of SFN, as well as the allyl-GSL of mustard seeds, which may have also contributed to the final biological scope. The largest zone of inhibition was reported for *Bacillus cereus*, but additional antimicrobial activities were observed for other Gram-positive bacteria, such as *Staphylococcus aureus* and *Listeria monocytogenes*, as compared to Gram-negative bacteria, such as *Salmonella enterica*. Additionally, strong inhibitory effects against *Salmonella typhimurium* strs. 10 and 30, resistant to antibiotics, *Escherichia coli* K12 (1 mg/mL, complete inhibition), and *Helicobacter pylori* were observed [107]. While the antimicrobial activity of broccoli extracts was comparable to that of ampicillin, chloramphenicol, tetracycline, and gentamicin, it was found that modified broccoli soup in the ileum might not have sufficient antimicrobial power at the colonic level, despite it being within the inhibitory range. This contradictory information could be due to the absorption/excretion backflow through enterohepatic circulation that would augment the frequency of derivatives from the mercapturic pathway, thus reducing the concentration of the actual bioactive unesterified SFN. In any case, from these results, it seems that bioactive organosulfur compounds of Brassica provide a valuable contribution towards the inhibition of the growth of bacteria present in the stomach and upper small intestine, as demonstrated by this dietary intervention, thus preventing the incidence of diverse intestinal inflammatory processes associated with some pathological bacterial strains [107].

In another study, the meal of 16 healthy subjects was supplemented with broccoli seed and sprout extracts (BSE, plus myrosinase, Avmacol^®^, Nutramax Laboratories Consumer Care Inc., Edgewood, MD, USA) to test for the possible effect of proton pump inhibition when taking omeprazole (Table 4) [87,88]. The primary hypothesis examined in this study was whether the stomach acidity would affect the activity of the myrosinase enzyme, co-delivered with GR into the intestinal lumen from Brassica-food material. In a previous encapsulation study, there was a 28% reduction in the conversion of GR to SFN between the coated tablets as compared to the uncoated tablets before taking omeprazole [87,88]. At the same time, the contribution of active myrosinase produced an average conversion of 36% regardless of the use of omeprazole. In the case of uncoated tablets before taking omeprazole, a high acidity was maintained, and its average conversion was therefore 25.7% [108]. An exhaustive genetic study of twenty genes that seemed to be potential pharmacodynamic biomarkers for SFN was carried out by real-time PCR: NQO1, NAD(P)H quinone oxidoreductase-1; *GCLC*, glutamate-cysteine catalytic ligase subunit; *GCLM*, glutamate-cysteine ligase modifying subunit; *HSP27* and *HSP70*, heat shock proteins 27 and 70, *HO-1*, heme oxygenase-1; *HDAC3*, histone deacetylase-3; *IL-1β*, interleukin-1β; *SOD2*, superoxide dismutase-2; *IL-2*, interleukine 2; *IL-10*, interleukine 10; *IL-6*, interleukine 6; *IL-8*, interleukine 8; *COX-2*, cyclooxygenase 2; *SLC7A11*, xCT, cysteine/glutamate antiporter; *IFNγ*: interferon-gamma; *CAT*, catalase; *AKR1c1*, aldo-keto reductase c1 family 1 member; and *AKR1B10*, aldo-keto reductase family 1 member b10, among others. These genes were selected based on their ability to upregulate transcription factor Nrf2 and heat shock response (HSR), and to inhibit the inflammatory pathway related to NF-IB [23,115,116,117,118]. Biomarkers of inflammation and immune response decreased in parallel to the increase in the bioavailability of SFN, while biomarkers of cytoprotective, detoxification, and antioxidant responses increased significantly. Furthermore, it is noteworthy that the level was extraordinarily high for IL-1β, IL-8, and COX2. All of these findings allowed concluding that the bioavailability of SFN from coated GRA-rich BSE, which also contains active myrosinase, was improved by 28% in subjects who had normal gastric acidity, while no changes were observed when subjects were under omeprazole treatment. However, adverse effects observed with coatings should be considered [108].

In a striking study, once-daily broccoli seed and sprout extract tablets produced by Avmacol^®^ (equivalent to 34 μmol GR, calculated to produce at least ~15 μmol SFN) with active myrosinase, were administered for potential disorder-relevant benefits of the autism spectrum (ASD) (Table 4). The clinical trial (based on previous evidence with positive effects on the behavior of young men and changes in urinary metabolomics in children with ASD) was a 15-week intervention study, randomized, parallel, double-blind, and placebo-controlled with a 15-week open-label treatment and 6-week treatment-free extensions in 57 children (3–12 years) with ASD [109]. The plasma levels of SFN metabolites showed considerable variability due to the timing of phlebotomy relative to SFN administration (3 to 8 h), as well as individual variation in metabolism. There was no statistically significant difference between the placebo (PL) and SFN groups when both took them at 22 and 30 weeks, returning to baseline at 36 weeks (after 6 weeks without SFN). Due to the importance of cellular oxidative stress and mitochondrial function in ASD, the authors measured reduced, oxidized, and total free glutathione (fGSH, fGSSG, and tGSH, respectively), showing the SFN treatment to be associated with lower ratios of both fGSH/fGSSG and tGSH/fGSSG [109]. This study also reported various cytoprotective gene products regulated by Nrf2, a master regulator of cellular redox homeostasis and an inhibitor of a key pro-inflammatory pathway [119], critical factors in this neuropathology. After 15 weeks of SFN treatment, among the Nrf2-dependent enzymes tested, the gene expression of *HO-1*, an essential enzyme in heme catabolism, was found to be significantly lower in the SFN-intake group compared to the PL-group. Increased levels of *HO-1* expression were consistent with decreases in proinflammatory cytokines. Regarding the gene expression of the cytoprotective heat shock protein HSP70, after 15 weeks, it was significantly lower in children taking SFN as compared to the PL group. Regarding the inflammatory indicators analyzed (previously known and characterized as biomarkers that are elevated in children with ASD [120]), children who took SFN for 15 weeks showed a significantly lower gene expression of IL-6 and TNF-α as compared to those belonging to the PL group; and changes in IL-1β expression levels were significantly greater as a result of the SFN intake than in the PL group, but not from baseline to week 15 or 22. The ATP-Linked respiration increased in individuals treated with SFN and was associated with changes in mitochondrial function, which is itself related to the enhanced ability of mitochondria to handle oxidative stress. Larger effects were observed in a subsample of children with severe ASD (OACIS-I scale). In addition, SFN appeared to improve socialization as oxytocin and improved other core features of ASD similar to bumetanide, but with the difference that, as a “natural” dietary component, SFN may have less potential for toxicity than any of the drugs with the long-term use, both for safety and long-term efficacy [109].

In another clinical study, seven women and three men were enrolled in a three-phase, double-blind, randomized crossover trial, each involving the consumption of 300 g of a broccoli soup made with one of three selected broccoli genotypes: Myb28B/B, Myb28B/V, and Myb28V/V (Beneforté^®^ (Seminis Vegetable Seeds, Inc., Arroyo Grande, CA, USA)), a broccoli variety that contains high levels of GR (10.68 µmol/g broccoli powder) [107,114]. With these GR-rich varieties, broccoli soups Myb28V/V and Myb28B/V were found to contain 452 and 280 µmol GR per 300 mL serving, respectively. These concentrations were approximately five and three times higher than Myb28B/B soup (84 µmol GR per 300 mL). An incidence of 40% of the null genotype for the enzyme glutathione *S*-transferase (*GSTM1*) was also determined in participants, which is within the expected range for Caucasians [121], but without a significant influence in this study. Glucoraphanin was detected in plasma and urine samples, as well as GE in urine; their cumulative amounts increased up to 8 h after consumption. The pharmacokinetics of the cumulative amount of SFN (35% unconjugated in plasma) and metabolites excreted in urine also indicated that it was significantly higher after consumption of Myb28V/V and Myb28B/V soups. It is worth mentioning that inter-individual differences in excretion were found, as already observed in many of the previously discussed studies. Several studies suggest that people who consume more than four to five servings of cruciferous vegetables per week have a reduced risk of multisite cancer and other chronic diseases [122,123,124]. Thus, the objective of this study was to quantify the pharmacokinetics of SFN derived from these new broccoli genotypes in the absence of any plant myrosinase activity. It was also observed that the improvements obtained concerning previous studies might be because the soups used were rich in fat and other nutrients that may facilitate the greater release of GR from the plant tissue in the gastrointestinal tract. Consistent with other findings, the most abundant metabolites excreted in urine were sulforaphane-NAC and erucin-NAC. The study provided evidence that the content of bioactive compounds is improved by selecting these genotypes, so that health benefits can be obtained with minimal changes in eating habits [99].

Beyond the clinical trials described in the present work, nowadays, there is a range of clinical trials (ClinicalTrials.gov Database (https://www.clinicaltrials.gov, accessed on 14 March 2023)) focused on the evaluation of different cruciferous-based foods and ingredients to discover the actual biological benefits in vivo (*n* = 28). These trials, which are at the recruiting phase, are mainly focused on dietary interventions aimed at shedding light on the biological relevance of ITC of Brassica foods (mainly SFN), concerning a diversity of pathological conditions, namely cancer, diabetes mellitus, or neurological disorders (autism), and also to establish the bioavailability and metabolism of different bioactive organosulfur compounds. Consequently, in the coming years, new interesting information on the biological scope of these compounds will be reported, and as a result, the preventive application of these plant-based foods will be fine-tuned as well as the monitoring capabilities according to the newly described biomarkers within the frame of the referred clinical trials.

## 7. Conclusions and Future

Through this review of recent studies, we have provided an overview of the potential of glucosinolate-hydrolysis products and their biologically formed metabolites, with clinical relevance in a broad spectrum of health problems. This information leaves us with various points to consider during the design and preparation of further investigations such as those highlighted here, with a major focus on the prevention and management of different (chronic) pathologies through diet, as well as in the decision-making processes for designing future studies aiming to demonstrate the health-promoting capacity of the bioactives found in Brassica foods. The expected impacts of these investigations will include recommendations for incorporating various portions of cruciferous vegetables (e.g., fresh sprouts, broccoli florets, mustard greens, microgreens, etc.) every week, and this is not only positive for a more balanced diet, but also because of the potential benefits beyond the supply of nutrients.

There will be key steps that need to be taken to achieve these milestones. On the one hand, we must seek the generation of knowledge that would improve our understanding of the clear relationship between specific bioactive compounds/metabolites—and their expected function. This new evidence will be paired with plausible impacts on measurable and clinically relevant outcomes to facilitate the preparation of recommendations for consumption. These improvements are of special interest when considering the potential of foods in the management of chronic conditions (e.g., non-communicable diseases, linked to metabolic problems).

On the other hand, work must be intensified for evaluating how the processing (either at industrial and/or household level) will affect the quality and dosage of bioactives from the given vegetable product, and the potential of synergies with combinations of specific vegetables (and/or ingredients) to facilitate the bioaccessibility of ITC, in the context of dietary-compatible consumption.

It is also interesting to discover, concerning the limiting step of the myrosinase and myrosinase-like activities in the gut microbiota, how to counteract the negative effects of mechanical and/or thermal degradation, and losses of activity, as well as other physical or chemical parameters that would affect its function. The study of the presence of myrosinase in food (plant tissue) and the large intestine is a long path to travel, with different paths for improving our current knowledge, to improve the bioavailability of ITC. The resulting greater fraction of delivered ITC will have an increased potential for improving the target organ, and exert higher functionality. The pathways and mechanisms through which GSL/ITC and their derivatives may exert a positive anti-inflammatory and antioxidant effect are growing in importance for many chronic conditions. The advances in the knowledge of biosynthetic routes, including the detection and analysis at the level of markers and genes involved, including exhaustive mapping, will facilitate the connections and also help in finding the best explanation for these processes from the plant tissue to the host metabolism.

The reviewed clinical studies set a precedent in a field of multiple and promising possibilities, in many cases without pre-existing studies, towards the nutritional improvement of our diet by incorporating more cruciferous vegetables, and also for explicit health purposes. There is a wide range of clinical applications incorporating cruciferous foods with preventive, palliative, or restorative purposes. It is worth mentioning that the studies including sprouts are of major interest, as they are fresh, edible, and naturally-rich sources of GSL (ITC), followed in the degree of importance by interventions with fresh mature plants (e.g., broccoli florets, cabbages, etc.). Lastly, although somewhat the less interesting, from the point of view of the diet purposes, by studies including industrial formulations (e.g., ingredients, nutraceuticals, etc.), not only because of the lower bioavailability but because of the paradigm of considering foods as the best possible vehicle in our diets for incorporating bioactive compounds, when possible, according to the availability of produce and acceptability of the consumers, which also involves also cultural aspects.

In future studies with volunteers and patients, the possibilities of using different cruciferous foods (combinations for better bioactive cocktails) must be explored, because modern analytical techniques available and implemented would help evaluate compounds and metabolites with much better accuracy than in previous years. Lastly, there is also a plethora of health problems to combat, where ITC and their metabolites can be used against many health problems such as: cancer (different types and in different organs), intestinal inflammation and conditions, obesity and adiposity, all kinds of inflammatory diseases, skin health, even in cognitive decline and certain illnesses, such as those within the autism spectrum.

The influence of gut microbiota and the microbiome of the digestive system affects all the processes in which ITC can play a positive role. This, together with BMI, different ages, and specific nutritional needs, have opened strong lines of research for personalized nutrition, and for studies on the influence of different human physiological states of development or conditions on health.

This review summarizes recent studies that provide interesting contributions, but also showed us the many potential venues for future research on the benefits of consuming cruciferous foods on our health and well-being, some of which are highlighted in these conclusive remarks. The research will continue to explore how cruciferous foods can be considered for multiple preventive and active programs in nutrition and wellness.

## Figures and Tables

**Table 1:** Basic chemical structure of those glucosinolates (GSL) analyzed in the frame of intervention studies or clinical trials and the association between precursor amino acids and individual GSL with detail of the chemical structure of the side chain (R).

Basic Chemical Structure					
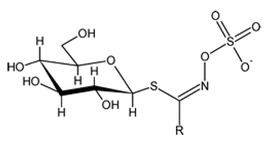					
Glucosinolate	Chemical Name	Side Chain (R)		Aminoacid Precursor	Reference
		Molecular Formula	2D Structure		
Aliphatic glucosinolates					
Alkenyl					
Gluconapin	3-butenyl-GSL	C_4_H_7_	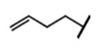	methionine (Met)	[9,10,11]
Glucobrassicanapin	4-pentenyl-GSL	C_5_H_9_	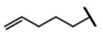	Met	[9,10,11]
Sinigrin	2-propenyl-GSL	C_3_H_4_	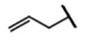	Met	[9,10,11]
Hydroxyalkenyl					
Progoitrin	2-hydroxy-3-butenyl-GSL	C_4_H_6_	** 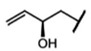 **	Met	[9,10,11]
Epiprogoitrin	2(S)-2-hydroxy-3-butenyl-GSL	C_4_H_6_	** 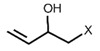 **	Met	[10,11,12]
Gluconapoleiferin	2-hydroxy-4-pentenyl-GSL	C_5_H_8_	** 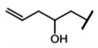 **	Met	[9,10,11]
Sulfur containing					
Glucoiberverin	3-methyltiopropyl-GSL	C_3_H_14_S	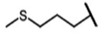	Met	[9,10,11]
Glucoerucin	4-methylthiobutyl-GSL	C_4_H_16_S	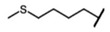	Met	[9,10,11]
Dehydroerucin	4-methylthio-3-butenyl-GSL	C_4_H_14_S	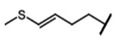	Met	[9,10,11]
Glucoiberin	3-methylsulfinylpropyl-GSL	C_3_H_12_SO	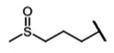	Met	[9,10,11]
Glucoraphanin	4-methylsufinylbutyl-GSL	C_4_H_14_SO	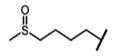	Met	[9,10,11]
Glucoalyssin	5-methylsulfinylpentyl-GSL	C_5_H_16_SO	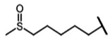	Met	[9,10,11]
Glucoraphenin	4-methylsulfinyl-3-butenyl-GSL	C_4_H_12_SO	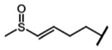	Met	[9,10,11]
Glucoerysolin	4-(methylsulfonyl)butyl-GSL	C_6_H_12_SO_2_	** 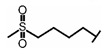 **	Met	[9,10,11]
Indolic glucosinolates					
Glucobrassicin	3-indolylmethyl-GSL	C_9_H_9_N	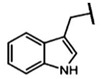	Tryptophan (Trp)	[9,10,11]
4-Hydroxy-glucobrassicin	4-hydroxy-3-indolylmethyl-GSL	C_9_H_9_NO	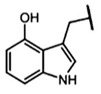	Trp	[9,11]
4-Methoxy-glucobrassicin	4-methoxy-3-indolylmethyl-GSL	C_10_H_11_NO	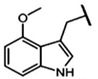	Trp	[9,10,11]
1-Methoxy-glucobrassicin	1-methoxy-indolylmethyl-GSL	C_10_H_11_NO	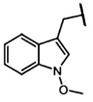	Trp	[9]
Neoglucobrassicin	*N*-methoxy-3-indlymethyl-GSL	C_10_H_11_NO	** 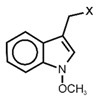 **	Trp	[10,11,13]
Phenyl (aromatic) glucosinolates					
Glucotropaeolin	Benzyl-GSL	C_7_H_8_	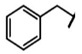	Phenylalanine (Phe)	[9]
Gluconasturtiin	2-phenetyl-GSL	C_8_H_10_	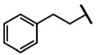	Phe	[9,10,11]
Sinalbin	4-hydroxybenzyl-GSL	C_7_H_8_O	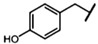	Phe	[9]

GSL, glucosinolate; Met, methionine; Phe, phenylalanine; Trp, tryptophan.

## Data Availability

Not applicable.
